# Granulocyte-colony stimulating factor for stroke treatment: mechanisms of action and efficacy in preclinical studies

**DOI:** 10.1186/2040-7378-1-2

**Published:** 2009-10-21

**Authors:** Jens Minnerup, Sevgi Sevimli, Wolf-Rüdiger Schäbitz

**Affiliations:** 1 Department of Neurology, University of Münster, Albert-Schweitzer-Straße 33, 48149 Münster, Germany; 2 Department of Neurology, University of Münster and Evangelisches Krankenhaus Bielefeld, Burgsteig 13, 33617 Bielefeld, Germany

## Abstract

G-CSF is widely employed for the treatment of chemotherapy-induced neutropenia. Recently, neuroprotective effects of G-CSF in animal stroke models were discovered including infarct size reduction and enhancement of functional recovery. The underlying mechanisms of action of G-CSF in ischemia appear to be a direct anti-apoptotic activity in neurons and a neurogenesis inducing capacity. Additional effects may be based on the stimulation of new blood-vessel formation, the stimulation of immunocompetence and -modulation as well as on bone marrow mobilization. In addition to a discussion of these mechanisms, we will review the available preclinical studies and analyze their impact on the overall efficacy of G-CSF in experimental stroke.

## Introduction

Granulocyte-colony-stimulating factor (G-CSF) was identified among a set of humoral factors on which the survival, proliferation, and differentiation of hematopoietic cells in cell culture assays is dependent [1,2]. After purification of the murine G-CSF more than 25 years ago its human analogue was discovered [1]. The complete species cross-reactivity [3] of the human and the murine G-CSF molecule exhibits a strong evolutionary conservation and emphasizes its importance for white blood cell regulation. A decade after its identification, G-CSF was approved by the FDA for prevention and treatment of chemotherapy-induced neutropenia and apheresis for hematopoietic transplantation [4,5]. Much interest focused on the use of G-CSF as a neuroprotective candidate when its infarct size reducing capabilities in animal stroke models were discovered in the year 2003 [6-8]. Beyond its initially as key protective mechanism assumed capability to mobilize bone marrow stem cells, a deeper understanding of G-CSF's action in stroke pathophysiology has been developed. This review focuses on the neuroprotective and neuroregenerative properties of G-CSF in animal models of focal cerebral ischemia. In addition, the evidence and efficacy from preclinical studies as the basis for current clinical trials is reviewed.

### Mechanisms of action of G-CSF in ischemic injury

#### Mobilization of stem cells

G-CSF's natural function of mobilizing stem cells from the bone marrow triggered initial explorations of its potential usefulness in stroke with the idea that mobilized stem cells may home into the injured brain [9]. A series of preclinical investigations in animals using G-CSF for the therapy of ischemic stroke was initiated to answer the question whether mobilized bone marrow cells contribute to improved outcome [9-11]. The capacity of bone-marrow derived cells to restore function in the injured brain has indeed been demonstrated (for review see [12]), but the mechanism of their advantageous action remains unclear. The proposed transdifferentiation of bone marrow derived cells into neural cells that induce functional and structural recovery poststroke was recently doubted by several studies (e.g. [13,14]). The assumption that G-CSF mobilized bone marrow cells might have caused the observed functional improvements was also propagated by Shyu et al [11]. However, dividing cells in the ischemic hemisphere, mainly seen in the subventricular zone, were presumably originated from adult neural stem cells concordantly with results from other groups [7,10]. Komine-Kobayashi and colleagues [15] subjected chimeric mice with EGFP-expressing bone marrow-derived cells to transient occlusion of the middle cerebral artery. The authors report that indeed migration of bone-marrow derived monocytes was not increased at all after G-CSF treatment, but rather decreased. So far not evidence proven is another bone marrow cell mediated mechanism. G-CSF may induce invasion of bone-marrow-derived stem cells into the infarcted brain which could contribute to enhanced neuro- and angiogenesis by secretion of neurotrophines and other trophic factors [12]. In conclusion the recent evidence from animal experiments cast doubt on the perception that mobilization of stem cells is the sole or even most important mechanism of action for functional recovery after G-CSF treatment.

#### Anti-apoptotic activity

**Figure 1:**
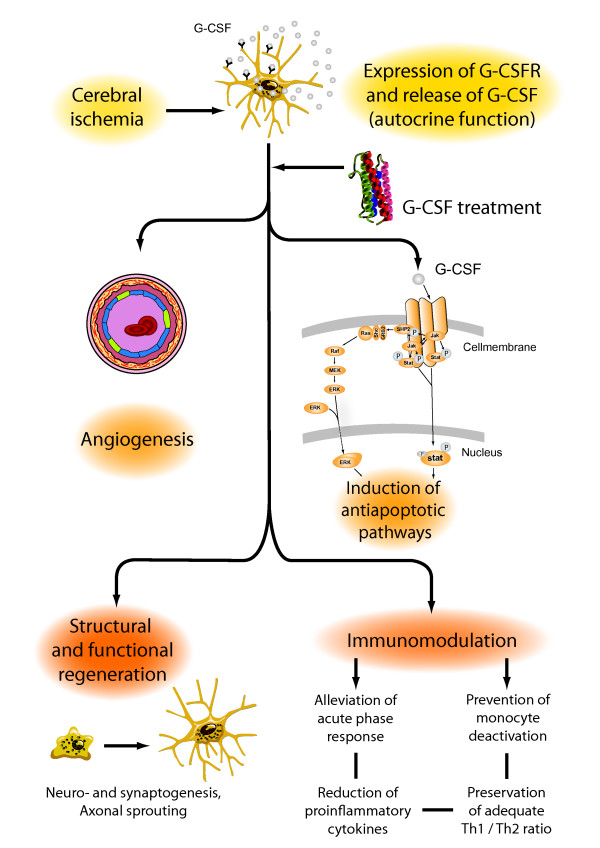
**G-CSF reduces infarct sizes and enhances functional recovery in stroke models by several mechanisms of action, such as the induction of anti-apoptotic pathways, neurogenesis and angiogenesis**. Thereby G-CSF acts as a direct protectant for neurons expressing its receptor. G-CSF's influences on immunocompetence and inflammation parameters are potential additional effects.

A first indication of a potential direct effect on cells of the brain came from the observation that G-CSF had a direct protective effect in cultured neurons against glutamate-induced cell death [6]. After cerebral ischemia, endogenously released G-CSF is presumably active on the upregulated G-CSF receptor in periischemic regions at risk, the so called penumbra, and may provide protection against apoptotic cell death in neurons (figure 1). Schneider showed that after interaction with its receptor, G-CSF activates through JAK signalling, three independent anti-apoptotic pathways: The signal transducer and activation of transcription (STAT)-3, the extracellular-signal-regulated kinase (ERK) and the phophatidylinositol 3-kinase (PI3K)-Akt pathway [7]. Komine-Kobayashi also found antiapoptotic effects of G-CSF on neurons after cerebral ischemia through the JAK/STAT signaling pathway and subsequent activation of Bcl-2 [15]. Moreover, G-CSF increased cIAP2 levels in the ischemic cortex and thereby decreased the activation of caspase 3, an important trigger of apoptotic processes [16]. In a rat model of intracerebral hemorrhage G-CSF's antiapoptotic activity in cells in the perihematomal area was revealed by a TUNEL assay, which detects less DNA fragments as a result from apoptotic signaling cascades after G-CSF treatment [17].

#### Neurogenesis

Neural progenitor cells reside for a lifetime in certain areas of the brain, particularly the subventricular zone (SVZ), the olfactory bulb and the hippocampus. Certain conditions such as stroke induce the generation of new neurons from precursor cells, a phenomenon which may potentially be utilized to restore brain function. G-CSF's most striking effect regarding neurogenesis was seen in the dentate gyrus, where the number of newly generated neurons under ischemic conditions [7,10,11] but also in nonischemic, sham-operated animals was increased. In the striatum there was only a trend toward an enhanced neurogenesis after G-CSF treatment which was not statistically significant and the number of newly generated cells was rather small [7]. This finding is not surprising, since the striatum is known to habor only a low number of neuronal precursors [18,19]. Generation of new differentiated cells from endogenous stem cells is an intricate interplay among different components such as proliferation, differentiation, and selective survival. In vivo experiments revealed that G-CSF promotes neurogenesis in all of these components. The number of newly generated cells was increased, the cells differentiate towards a neuronal fate and anti-apoptotic pathways are activated [7]. The in vivo findings of an increased neurogenesis after G-CSF treatment were confirmed by in vitro experiments. It was shown that adult neural stem cells isolated from the rat SVZ or hippocampal region that grow as neurospheres in culture express the G-CSF receptor [7,20]. G-CSF dose-dependently induced maturation of cultured progenitor cells towards a neuronal phenotype and increased the population of the differentiated cells [7].

#### Angiogenesis

Angiogenesis is a process where new vessels arise from pre-existing ones [21]. Future treatment strategies in stroke focus on optimisation of this process in the ischemic boundary zone [22]. However, the contribution of angiogenesis to functional recovery after stroke is still unclear [23-25]. Lee and colleagues showed that G-CSF enhanced angiogenesis in a rat stroke model measured by endothelial cell proliferation, the vascular surface area, the number of branch points, and the vascular length [26]. The G-CSF effect was more pronounced when treatment was initiated earlier. But even when treatment was delayed up to seven days after the induction of ischemia an increased angiogenesis accompanied by an enhanced long-term functional recovery could be observed [26]. Expression of the vascular marker von Willebrand factor in BrdU positive cells after G-CSF treatment demonstrated the generation of new endothelial cells [11]. Taguchi found an accelerated angiogenesis measured by an angiographic score without an enhanced functional outcome [27]. However, the results of this study have to be interpreted with caution since immunodeficient mice were used in which G-CSF may not exert its immunomodulative properties. The immunomodulative effects are presumably important for post-stroke functional recovery, as described below.

#### Immunomodulation

Recent research revealed that interactions between cerebral ischemia and the immune system are exceptionally relevant for the functional outcome of stroke patients [28]. Stroke induced immunodepression can cause infections, such as pneumonia, a frequent complication in stroke patients. However, immunodepression may potentially improve stroke outcome by alleviating the autoaggressive responses due to ischemia-induced exposure of central nervous system-specific antigens to the immune system [29-31]. Thus, immunomodulation and an increase of in immunocompetence may also be responsible for the rather acute effects of G-CSF[32] Indeed, a reduced infiltration of neutrophils and microglia in the ischemic hemisphere after G-CSF treatment was observed [15,26], whereas our group could not detect such a difference between the placebo group and the G-CSF group [6]. A further analysis of inflammatory cells in the ischemic hemisphere revealed a decreased activation of inducible nitric oxide synthase (iNOS)-positve microglia in animals treated with G-CSF [15]. As a consequence of the iNOS inhibition, a reduced nitrotyrosine production as a marker for nitrosadative stress was detected in NeuN positive cells [15]. In contrast to these immunohistochemistry and western blot findings there was no G-CSF induced reduction of iNOS on the mRNA level [33]. Administration of interleukin-1 beta is known to deteriorate cerebral ischemia and an interleukin-1 beta receptor antagonist may neutralize this effect [34]. Thus, the reduction of the ischemia induced interleukin-1 beta upregulation by G-CSF may contribute to infarct size reduction [16,33]. Recently, our group showed that G-CSF suppresses MMP-9, which is known to mediate inflammation, blood-brain barrier breakdown with subsequent edema formation and tissue injury in acute stroke [35].

### Efficacy of G-CSF in stroke models

#### G-CSF in experimental stroke and the STAIR criteria

Successful testing of a candidate stroke drug in animal models does not firmly predict efficacy in clinical studies [36]. As a result of many failed clinical stroke trials the *S*troke *A*cademic *T*herapy *I*ndustry *R*oundtable (STAIR) established recommendations for the preclinical evaluation of stroke drugs [37]. The STAIR criteria postulate that the efficacy of a new drug should be demonstrated in a variety of stroke models performed in different species and by different laboratories. Indeed, G-CSF showed efficacy in different species and different stroke models such as transient ischemia in mouse [9,15,38,39] and rat [6,7,11] as well as permanent ischemia in mouse [10,38] and rat [7,8]. Moreover, G-CSF's efficacy was investigated in animals with comorbitiy, such as diabetes and hypertension, which is important, since stroke patients usually exhibit those conditions [40,41]. As recommended by the STAIR functional outcome in animal should be tested besides measuring infarct size reduction. G-CSF demonstrated an improvement in short-term [7,15,38] and long-term [7,8,10,11,39] functional neurological deficits. Systemic parameters relevant for stroke pathophysiology such as blood pressure or oxygen saturation were not influenced by G-CSF as measured by physiological monitoring of animals subjected to stroke [6,7,15]. The overall high methodological quality of preclinical G-CSF stroke studies was corroborated by a recent systemic analysis [42]. Philip and colleagues found that animal experimental stroke studies of G-CSF had the highest quality in a STAIR guideline derived quality score compared to all other neuroprotective agents that are currently investigated in clinical phase II or III trial [42].

#### Meta-analysis and meta-regression analysis of G-CSF in experimental stroke

To enhance the chance of a successful transfer of preclinical data in clinical trials beyond the application of the STAIR criteria, systematic meta-analyses of candidate neuroprotectants in animal experiments were conducted [43-46]. To get an overall impression of G-CSF's efficacy in the recently published preclinical studies and for potential guidance of further clinical studies, we have performed a meta-analysis and meta-regression analysis of G-CSF in animal models of focal cerebral ischemia [47]. The meta-analysis showed that G-CSF effectively reduced both infarct volumes and sensorimotor deficits. Infarct sizes were reduced by 42%. The reduction of infarct volumes in G-CSF-treated animals was proportional to the infarct volumes of placebo-treated animals as indicated by the L'abbé plot [47]. This proportional infarct size reduction demonstrates G-CSF's efficacy in milder stroke models as well as in severe hemispheric stroke models. Sensorimotor deficits which were categorized in three subgroups (Rotarod running, neuroscore, limb function) were improved between 24% and 40%. Our meta-regression, which was the first meta-regression analysis of a neuroprotective drug in animal stroke models, identified higher doses of G-CSF to be associated with significantly smaller infarct volumes for doses between 10 and 60 μg/kg body weight (infarct size reduction 0.8% per one μg/kg body weight increase in dose when applied within the first 6 hours and 2.1% per one μg/kg body weight increase in dose when applied later than 6 hours after induction of ischemia). Time on Rotarod was significantly extended by 2.1% and 2.2% per one μg/kg body weight increase in dose for early and late treatment initiation, respectively. Also, limb function and neuroscore improved significantly when G-CSF dose was increased. This dose-response relationship is particularly important finding since conclusive experimental dose finding data deriving from a singular stroke study are currently not available. Also a critical aspect of stroke drug development is the therapeutic time window. For G-CSF effects on infarct size the time window is at least 24 hours in the transient suture occlusion model in rodents [9,11]. Regarding functional outcome Zhao [48] reported a beneficial effect of G-CSF when administered more than three month after the onset of ischemia. Using a meta-regression technique we found that a delayed treatment was as effective as an early treatment initiation and may even lead to smaller infarct sizes [47]. This result is particularly interesting since the time window for most candidate neuroprotectants closes early after symptom onset [49]. The potential of a much longer time-window of G-CSF compared to other stroke drugs might be explained by the above mentioned multimodal actions consisting of neuroprotective and particularly proregenerative properties.

## Conclusion

Hematopoietic factors as candidate drugs for stroke treatment were intensely studied in stroke models over the last years. However, efficacy of candidate neuroprotectants in animal experiments may not necessarily predict efficacy in stroke patients, particularly when the preclinical experiments are insufficient and incomplete. New candidate drugs should therefore be tested in stroke models threefold to enhance the chance of a successful bench-to-bedside progress: 1. Meaningful interaction in stroke pathophysiology, 2. Integrity regarding fulfilled STAIR criteria, and 3. Efficacy analyzed in meta-analysis of animal studies. G-CSF, as novel candidate stroke drug, widely addresses these issues due to its multimodal mode-of-action in combination with a broad spectrum of efficacy in animal stroke models. Aside from this, G-CSF's has a comprehensive safety profile as demonstrated by its clinical use for many years.

## Abbreviations

G-CSF: Granulocyte-colony stimulating factor; STAIR: Stroke Therapy Academic Industry Roundtable; EGFP: enhanced green fluorescent protein; STAT: signal transducer and activation of transcription; ERK: extracellular-signal-regulated kinase; PI3K: phophatidylinositol 3-kinasel; Bcl-2: B-cell lymphoma 2; TUNEL: terminal uridine deoxynucleotidyl transferase dUTP nick and labeling; SVZ: subventricular zone; BrdU: bromdeoxyuridine; iNOS: inducible nitric oxide synthase.

## Competing interests

WRS is inventor on a patent application regarding the neuroprotective effects of G-CSF.

## Authors' contributions

JM wrote the manuscript. SS wrote the manuscript. WRS supervised manuscript preparation.
